# Developing clinical prediction models when adhering to minimum sample size recommendations: The importance of quantifying bootstrap variability in tuning parameters and predictive performance

**DOI:** 10.1177/09622802211046388

**Published:** 2021-10-08

**Authors:** Glen P Martin, Richard D Riley, Gary S Collins, Matthew Sperrin

**Affiliations:** 1 Division of Informatics, Imaging and Data Science, Faculty of Biology, Medicine and Health, 5292 University of Manchester, Manchester Academic Health Science Centre, UK; 2 Centre for Prognosis Research, School of Medicine, Keele University, UK; 3 Centre for Statistics in Medicine, Nuffield Department of Orthopaedics, Rheumatology and Musculoskeletal Sciences, University of Oxford, UK

**Keywords:** Clinical prediction model, penalisation, shrinkage, validation, overfitting

## Abstract

Recent minimum sample size formula (Riley et al.) for developing clinical prediction models help ensure that development datasets are of sufficient size to minimise overfitting. While these criteria are known to avoid excessive overfitting on average, the extent of variability in overfitting at recommended sample sizes is unknown. We investigated this through a simulation study and empirical example to develop logistic regression clinical prediction models using unpenalised maximum likelihood estimation, and various post-estimation shrinkage or penalisation methods. While the mean calibration slope was close to the ideal value of one for all methods, penalisation further reduced the level of overfitting, on average, compared to unpenalised methods. This came at the cost of higher variability in predictive performance for penalisation methods in external data. We recommend that penalisation methods are used in data that meet, or surpass, minimum sample size requirements to further mitigate overfitting, and that the variability in predictive performance and any tuning parameters should always be examined as part of the model development process, since this provides additional information over average (optimism-adjusted) performance alone. Lower variability would give reassurance that the developed clinical prediction model will perform well in new individuals from the same population as was used for model development.

## Background

Clinical prediction models (CPMs) aim to predict the risk of an event-of-interest occurring given an individual's set of predictor variables.^1,2^ CPMs have many practical uses in healthcare such as aiding in treatment planning, underpinning decision-support, or facilitating audit and benchmarking. To support such uses, the process of CPM development requires careful consideration, and has correspondingly received large attention in both the statistical and medical literature.^3–6^

A primary concern in prediction modelling is to ensure that the developed CPM remains accurate in new (unseen) observations. However, predictive accuracy of a CPM often drops between development and validation.^7,8^ Using data that have insufficient observations (i.e. small sample size) for CPM development often contributes to this reduction in predictive performance, and leads to models that are overfitted. Overfitting results in predicted risks that, on average, are too extreme in new individuals and thereby the model may not perform well at the time of model validation or implementation.

Sample size justification for CPM development studies was historically based on having an events per predictor parameter (EPP, also known as events per variable) of 10 or more.^9–11^ This rule-of-thumb has been shown to be overly simplistic and has weak evidence to support its use,^12^ with formal sample size formula recently proposed by Riley et al.^13–15^ Appealingly, the criteria outlined in these sample size formulae aim to reduce the potential for a developed CPM to be overfitted to the development data set.

Correspondingly, the use of penalisation methods, which reduce variance but introduce bias into parameter estimation through shrinking parameter estimates towards zero, have previously been recommended to develop CPMs in smaller sample sizes.^11,16–18^ Such techniques include LASSO regression, ridge regression and Firth's correction.^19–21^ Compared with unpenalised estimation methods (such as traditional maximum likelihood estimation, MLE), several studies have found that predictive performance can be improved through penalisation methods, especially when the EPP is small.^12,16,17,22^ Nevertheless, such methods do not themselves justify developing CPMs in data of insufficient size.^23^ Recent work by Van Calster et al.^24^ and Riley et al.^23^ found that while parameter shrinkage improved prediction accuracy on average, the between-sample variability of predictive performance metrics was high, especially in small EPP. Additionally, these studies found a negative correlation between the estimated shrinkage and the ‘true’ shrinkage, meaning that the level of penalisation was lower in scenarios where it was most needed; this finding supported earlier work by van Houwelingen.^25^

However, it remains uncertain whether the previously observed between-sample variability of predictive performance metrics persists in data that meet, or surpass, the recently proposed Riley et al. criteria.^13–15^ If such variability does persist, then examining this as part of the model development processes would be crucial; we aimed to investigate this concept here. In particular, it seems prudent to use penalisation methods to derive a CPM, but only in data that meet minimum sample size requirements.^13–15^ Theoretically, such a combined approach would expose the CPM to the benefits of penalisation, while avoiding development on insufficient data. For example, penalisation methods such as LASSO can aid in variable selection,^19^ while penalisation through a Bayesian perspective^26^ would allow the modeller to incorporate prior knowledge directly into the CPM derivation (e.g. from expert opinion or existing CPMs).^27^

Therefore, the aim of this study is two-fold. First, to examine the characteristics of CPM performance metrics, upon validation, of models developed using a range of penalisation methods compared with unpenalised maximum likelihood estimation, in derivation data that satisfy formal sample size criteria. Second, to explore the importance of quantifying variability in predictive performance as part of the model development processes, for example through bootstrap internal validation. We investigate these aims through a simulation study and real-world clinical example of critical care data. Note, we are interested in variability in overall performance, rather than stability of individual risks as studied recently.^28^

The remainder of the paper is structured as follows: section ‘Shrinkage and penalisation methods to developing prediction models’ describes the common approaches to develop CPMs using penalisation; section ‘Riley et al. sample size criteria’ gives a brief overview of the Riley et al. sample size criteria; section ‘Simulation study’ describes the methods and results of our simulation study; while section ‘Empirical study’ reports the results from the real-world critical care example. Finally, concluding remarks are given in section the ‘Discussion’ section.

## Shrinkage and penalisation methods to developing prediction models

Throughout, we consider the development of a CPM to estimate the probability of a binary outcome, *Y*, conditional on a set of *P* predictors, which we denote $X_{1},\ldots ,X_{P}$ . We assume that we have $i=1,\ldots ,N_{dev}$ observations in a development dataset, where $N_{dev}$ is (at least) the minimum required sample size as determined by the Riley et al. sample size criteria.^13–15^ This development dataset is used to fit a logistic regression CPM of the form:

(1) $$P(Y_{i}=1)=g^{-1}(\beta_{0},\beta_{1},\ldots ,\beta_{P})={\left[1+\exp\left(-\left(\beta_{0}+\overset{P}{\underset{\;p=1}{\sum }}\;\beta_{p}X_{i,p}\right)\right)\right]}^{-1}$$

for logit-link-function, g() , and where the unknown parameters, $\beta_{0},\beta_{1},\ldots ,\beta_{P}$ , are log-odds, which are estimated within the development data using either unpenalised or penalised approaches to inference. In this paper, we consider the following estimation methods.

### Unpenalised maximum likelihood estimation (MLE)

This is the standard unpenalised approach to developing a logistic regression CPM, whereby the regression coefficients are estimated by maximising the following log-likelihood (LL) function:

$$LL(\beta_{0},\beta_{1},\ldots ,\beta_{P})=\overset{N_{dev}}{\underset{i=1}{\sum }}\;y_{i}\log(g^{-1}(\beta_{0},\beta_{1},\ldots ,\beta_{P}))+(1-y_{i})\log(1-g^{-1}(\beta_{0},\beta_{1},\ldots ,\beta_{P})).$$

### Closed-form uniform shrinkage

This approach applies a post-estimation uniform shrinkage factor to all the coefficients estimated using the unpenalised MLE approach, where the shrinkage factor is calculated based on the likelihood ratio statistic.^13,14,29^ The shrinkage factor (*S*) is calculated as

$$S=1+\frac{P}{N_{dev}\log\left(1-\left(1-\exp\left(\frac{-LR}{N_{dev}}\right)\right)\right)}$$

where LR is the likelihood ratio statistic of the fitted model (estimated by unpenalised MLE), compared with a null (intercept-only) model. The shrunken coefficients are then calculated by multiplying each of the regression coefficients estimated through MLE by *S* (i.e. $S\times \beta_{p}$ for p=1,…,P ). The intercept, $\beta_{0}$ is then re-estimated to ensure the overall outcome proportion is accurate.

### Uniform bootstrap shrinkage

This is similar to the closed-form uniform shrinkage, except that the shrinkage factor is calculated through the following steps: (i) take bootstrap samples from the development data, (ii) fit a (MLE) model in this bootstrap sample replicating *all* modelling steps, (iii) calculate the linear predictor of this model on each observation in the original development data and (iv) fit a logistic model to the observed outcomes with the linear predictor from step (iii) as the only covariate. In this study, we repeated this process 500 times and took the shrinkage factor to be the average of the corresponding coefficient from step (iv). In essence, this shrinkage factor is an estimate of the in-sample optimism of the calibration slope, as calculated using a bootstrap internal validation.^6,29^

### Firth's correction

Here, we implement bias-reduced penalised logistic regression, as proposed by Firth.^21^ This approach is equivalent to penalising the LL by a Jeffrey's prior of a logistic regression model. In particular, if we denote $I({\hat{\beta }}_{0},{\hat{\beta }}_{1},\ldots ,{\hat{\beta }}_{P})$ as the Fisher information matrix, then Firth's correction maximises a penalised LL of the form:

$$LL(\beta_{0},\beta_{1},\ldots ,\beta_{P})+\frac{1}{2}\log(\left|I({\hat{\beta }}_{0},{\hat{\beta }}_{1},\ldots ,{\hat{\beta }}_{P})\right|).$$

As with closed-form uniform shrinkage and uniform bootstrap shrinkage, the intercept, $\beta_{0}$ is re-estimated to ensure the overall outcome proportion is accurate, as suggested previously.^30^

### Penalised logistic regression using LASSO

LASSO penalises the (log-)likelihood of the logistic regression model, such that the coefficients are shrunk towards zero and some coefficients might be shrunk to exactly zero (thereby performing variable selection).^19^ Explicitly, LASSO maximises a penalised LL of the form:

$$LL(\beta_{0},\beta_{1},\ldots ,\beta_{P})-\lambda \overset{P}{\underset{\;p=1}{\sum }}\;|\beta_{p}|$$

where λ is a tuning parameter that controls the degree of penalisation. In this study, we selected λ using 10-fold cross-validation to minimise the deviance. We also considered repeated 10-fold cross-validation, whereby the 10-fold cross-validation procedure was repeated 100 times and we selected the λ that minimised the deviance averaged across the 100 replications (i.e. minimizes the averaged error curves across λ ).

### Penalised logistic regression using ridge

This approach is similar to LASSO, except that coefficients are shrunk towards zero but none will be exactly zero.^20^ Explicitly we maximise the penalized LL

$$LL(\beta_{0},\beta_{1},\ldots ,\beta_{P})-\lambda \overset{P}{\underset{\;p=1}{\sum }}\;\beta_{p}^{2}$$

Again, we selected λ using both single 10-fold cross-validation to minimise the deviance, and repeated 10-fold cross-validation.

## Riley et al. sample size criteria

In this section, we give an overview of the sample size criteria proposed by Riley et al.^13–15^ However, we refer readers to previous publications^13–15^ for a detailed explanation and example illustrations for how to calculate these criteria.

The Riley et al. criteria^13^ for calculating minimum sample sizes for logistic regression CPMs are based on satisfying all of the following criteria: (i) a uniform shrinkage factor of >0.9, (ii) ensuring a small absolute difference in the apparent and adjusted Cox-Snell R-squared, $R^{2}$ and (iii) ensuring a precise estimate of the model intercept. For example, the required minimum sample size required to satisfy criteria (i) can be calculated by

$$N_{dev}=\frac{P}{(S-1)\log\left(1-\frac{R^{2}}{S}\right)}$$

where *P* is the number of candidate predictor variables, *S* is the pre-specified required maximum level of shrinkage (e.g. usually set to 0.9), and $R^{2}$ is the pre-specified anticipated Cox-Snell $R^{2}$ . Hence, to calculate the minimum sample size required to meet these criteria, one needs to pre-specify a sensible value for the Cox-Snell $R^{2}$ . Where possible, Riley et al.^13^ recommend that this is based on existing prediction models developed for similar outcomes and similar populations. In this study, we consider two ways of doing this, which are outlined below in section ‘Data-generating mechanism and simulation scenarios’.

Mathematical details for criteria (ii) and (iii) are given in Riley et al.^13^ The minimum required sample size is then taken as the maximum required to meet criteria (i)–(iii). In this study, we used the ‘pmsampsize’^31^ R package to estimate the minimum required sample size.

## Simulation study

We now describe the design and results of our simulation study, which aimed to investigate the predictive performance of CPMs developed using MLE, post-estimation shrinkage (closed-form uniform shrinkage and uniform bootstrap shrinkage) and penalised regression (Firth's, LASSO and ridge) approaches (section ‘Shrinkage and penalisation methods to developing prediction models’), on data that meet minimum sample size requirements.^13–15^ We designed the simulation following best practice guidelines.^32^

### Data-generating mechanism and simulation scenarios

Throughout all simulations, we begin by generating a large (*N* = 1,000,000 observations) population-level dataset, which aims to mimic an overarching population that one subsequently obtains random samples from to develop a CPM. We generated P=10 predictors, each from a standard normal distribution. Additionally, each observation (reflecting an individual participant) had a binary outcome, *Y*, which we simulated conditional on their simulated predictor values according to the following data-generating model (based on equation (1)):

$$P(Y_{i}=1)={\left[1+\exp\left(-\left(\beta_{0}+\overset{10}{\underset{\;p=1}{\sum }}\;\beta_{p}X_{i,p}\right)\right)\right]}^{-1}.$$

Here, $\beta_{1},\ldots ,\beta_{10}$ represent ‘true’ log-odds ratios, which were varied across two different specifications. First, we considered scenarios where all ten covariates were truly associated with *Y*, such that $\beta_{1}=\ldots =\beta_{6}=\log(1.1)$ , $\beta_{7}=\beta_{8}=\log(1.5)$ and $\beta_{9}=\beta_{10}=\log(2)$ . Second, to consider situations where the data had more ‘noise’, we also considered scenarios where only the first five covariates where truly associated with *Y*, such that $\beta_{1}=\ldots =\beta_{3}=\log(1.1)$ , $\beta_{4}=\log(1.5)$ , $\beta_{5}=\log(2)$ and $\beta_{6}=\ldots =\beta_{10}=0$ . These values of $\beta_{1},\ldots ,\beta_{10}$ aim to mimic values that one might expect to find in ‘real data’. The functional form of all covariates were specified as linear (i.e. non-linear associations were not considered). Additionally, $\beta_{0}$ was chosen in each simulation to give an overall outcome proportion of either 20 % or 50 %, which was varied across simulation scenarios.

Following generation of this population-level data, we randomly sampled (without replacement) a development cohort of size $N_{dev}$ , to represent a dataset available to the analyst/ modeller to develop their CPM. The value of $N_{dev}$ was taken as the minimum sample size required to satisfy the criteria outlined by Riley et al.^13–15^ (section ‘Riley et al. sample size criteria’), and was calculated in each simulation iteration across all simulation scenarios. For these sample size calculations, we used the anticipated event proportion in a given simulation scenario (i.e. either 20% or 50%). Additionally, as outlined in section ‘Riley et al. sample size criteria’, one needs to pre-specify a sensible value for the Cox-Snell $R^{2}$ . In this simulation study, we considered two different approaches for pre-specifying the Cox-Snell $R^{2}$ . Firstly, we fitted a logistic regression model in the population-level data, that included all 10 covariates, and calculated the following Cox-Snell $R^{2}$ for use in the sample size calculations, as outlined in Riley et al.:^13^

(2) $$R^{2}=\left(1+\frac{P}{n\log\left(1-\left(1-\exp\left(\frac{-LR}{n}\right)\right)\right)}\right)\times \left(1-\exp\left(\frac{-LR}{n}\right)\right)$$

where, in this case, P=10 , n=1,000,000 , and LR is the likelihood ratio statistic of the full- and null-model in the population-level data.

Secondly, given that in practice such information might not be available *a priori*, we also considered the recommendation of Riley et al.^15^ to calculate

(3) $$R^{2}=0.15\times max(R^{2})$$

where $max(R^{2})=1-(\phi^{\phi }\times (1-\phi {)}^{1-\phi }{)}^{2}$ , with ϕ denoting the observed outcome proportion. This second approach corresponds to proposing a CPM that can explain 15% of the variance and is relatively conservative, as it leads to larger required sample sizes. Note, for logistic regression models with outcome proportions of 0.5, 0.4, 0.3. 0.2, 0.1, 0.05 and 0.01, the corresponding $max(R^{2})$ values are 0.75, 0.74, 0.71, 0.63, 0.48, 0.33 and 0.11, respectively.

The above data-generating processes were implemented across all combinations of $\left\{\beta_{1},\ldots ,\beta_{10}\right\}$ , outcome proportions (20% or 50%), and using equations (2) and (3) to pre-specify Cox-Snell $R^{2}$ in calculating $N_{dev}$ . This resulted in eight simulation scenarios, each of which were run across 500 iterations of the above data-generating processes. The simulation scenarios are overviewed in Table 1. The simulation scenarios aim to cover a range of possible model development settings, but we recognise this is not an exhaustive list.

**Table 1.** Overview of each simulation scenario.

Simulation scenario	Prevalence of Y	R2 for sample size calculation	Beta
1	0.2	Sample size based on population R2	All 10 predictors
2	0.2	Sample size based on population R2	5 predictors and 5 noise terms
3	0.5	Sample size based on population R2	All 10 predictors
4	0.5	Sample size based on population R2	5 predictors and 5 noise terms
5	0.2	Sample size based on max R2	All 10 predictors
6	0.2	Sample size based on max R2	5 predictors and 5 noise terms
7	0.5	Sample size based on max R2	All 10 predictors
8	0.5	Sample size based on max R2	5 predictors and 5 noise terms

### Methods considered

Within each sampled development cohort (of size $N_{dev}$ ), we fitted a logistic regression model using equation (1). The unknown parameters (i.e. $\beta_{0},\beta_{1},\ldots ,\beta_{10}$ ), were estimated under the following inference methods, each as described in section ‘Shrinkage and penalisation methods to developing prediction models’: (i) unpenalised maximum likelihood estimation (MLE), (ii) closed-form uniform shrinkage, (iii) uniform bootstrap shrinkage, (iv) Firth's correction, (v) penalised logistic regression using LASSO, and (vi) penalised logistic regression using Ridge. For (v) and (vi) we used both single 10-fold cross-validation and repeated 10-fold cross-validation.

### Performance measures

We quantified the predictive performance of each analysis model using calibration (agreement between the observed and expected outcome proportions, across the full risk range) and discrimination (ability of the model to separate those who have the outcome from those that do not have the outcome) within an independent validation set. This validation set was formed in each simulation iteration by including all observations from each simulated population-level dataset that were not sampled into the development cohort. This represents extremely large-sample independent validation (i.e. *N* = 1,000,000 minus $N_{dev}$ ), and helps ensure that the standard error of estimated performance metrics was low, and hence that any observed variability was due to sampling from the population-level data, rather than uncertainty in estimating the performance metrics.

In each of the samples from the validation set, calibration was quantified with the calibration-in-the-large and calibration slope. Calibration slope was estimated by fitting a logistic regression model to the observed outcomes in the validation data with the linear predictor of each analytic method as the only covariate, alongside an intercept. Calibration-in-the-large was obtained by the intercept estimate when fitting the same model but with the slope fixed at unity. A calibration-in-the-large less than 0 implies the model overestimates the overall outcome proportion in the validation data, while a calibration slope less than 1 implies model overfitting. Discrimination was quantified using the area under the receiver operating characteristic curve (AUC). Additionally, we estimated the Cox-Snell $R^{2}$ and the Brier score of each method within the validation set.

Alongside investigating the distribution of the estimated calibration-in-the-large, calibration slope, AUC, Cox-Snell $R^{2}$ and Brier score across the 500 iterations, for each estimation method, we also calculated the associated median and the 2.5–97.5% quantile to summarise average predictive performance. Root-mean-square deviation in the calibration-in-the-large and calibration slope was also calculated for each model by taking the square root of the mean squared-difference between the estimated calibration-in-the-large/calibration slope and the corresponding reference value (0 or 1, respectively) across the 500 iterations per simulation scenario.

### Software

R version 4.0.2 was used for all simulations,^33^ along with the packages ‘tidyverse’,^34^ ‘pROC’,^35^ ‘glmnet’,^36^ ‘logistf’^37^ and ‘pmsampsize’.^31^ The ‘glmnet’ package was used to fit the LASSO and Ridge models (using the default cross-validation selection procedure for λ ; that is, a grid of 100 λ values from 0.0001 to $\lambda_{max}$ – the smallest value for which all coefficients are zero), the ‘pmsampsize’ package was used to estimate the minimum required sample size based on Riley et al.^13^ and the ‘logistf’ package was used to fit logistic regression models with Firth's correction. All other code was written by the authors and is available via the first author's GitHub page (https://github.com/GlenMartin31/Penalised-CPMs-In-Minimum-Sample-Sizes), along with the full data on which the results of this simulation study are based. Note, we also re-ran the aforementioned simulations with a pairwise correlation of 0.5 between the 10 predictors; the results are quantitatively similar to those presented here, so we do not discuss these further (the data is available on the GitHub repository for exploration).

### Simulation results

#### Minimum required sample size

The minimum required sample sizes across simulation iterations are summarised in Supplemental Table 1, for all simulation scenarios (i.e. Table 1). For each of the simulation scenarios where the sample size calculation was based on equation (3) – that is, based on 15% of maximum Cox-Snell $R^{2}$ – the minimum required sample size was $N_{dev}=\;898$ for an overall outcome proportion of 20 %, and was $N_{dev}=749$ for an overall outcome proportion of 50 %. In all of these scenarios, the required sample size was driven by meeting criteria 1 of Riley et al. (i.e. a uniform shrinkage factor of >0.9 ).^13^

Supplemental Figure 1 shows the scatter of the Cox-Snell $R^{2}$ that was used within the sample size calculations, against the Cox-Snell $R^{2}$ achieved for the prediction models estimated by (unpenalised) MLE, upon validation, across simulation scenarios (similar results for each other estimation method). In all scenarios where the sample size calculation was based on 15% of maximum $R^{2}$ and all ten predictors were truly associated with *Y* (i.e. scenarios 5 and 7), the Cox-Snell $R^{2}$ of the derived CPMs (upon validation) was higher than that used to calculate the minimum sample size requirements in all 500 iterations. Supplemental Figure 1 shows that the reverse was true where the sample size calculation was based on the population $R^{2}$ , thereby representing cases where the developed CPMs never achieve what was expected *a priori* in terms of Cox-Snell $R^{2}$ .

#### Average performance upon validation

Table 2 shows the median (taken across the 500 iterations for each scenario) of the calibration slope, where we find that the median calibration slopes were close to 1 for all methods, but in absolute terms the median calibration slope was closer to 1 for uniform closed-form shrinkage, uniform bootstrap shrinkage, Firths correction, LASSO, and Ridge compared with unpenalised MLE. As expected, the calibration slope of the unpenalised MLE was >0.9, on average, for the scenarios where the pre-specified Cox-Snell $R^{2}$ used to calculate the minimum sample size was met (or surpassed) by the derived model – this is an expected property of the Riley et al. criteria.^13–15^ Any scenarios where the median calibration slope for the unpenalised MLE was slightly lower than 0.9 (e.g. 0.87–0.89) represent those where the where the Cox-Snell $R^{2}$ used to calculate the minimum sample size was not subsequently achieved by the model (see Supplemental Figure 1). This demonstrates the need to carefully consider how one pre-specifies the Cox-Snell $R^{2}$ when applying the sample size criteria. Supplemental Table 2 shows the corresponding results of the calibration-in-the-large for each analytical method, upon validation. In all cases, the median calibration-in-the-large was close to zero for all models, indicating accurate calibration-in-the-large (i.e. estimates of overall outcome proportion) on average.

**Table 2.** The median (2.5% and 97.5% quantile) of the calibration slope for each analytical method, upon validation, across the 500 iterations for each simulation scenario. See Table 1 for the numbering of each simulation scenario.

Simulation scenario	MLE	Uniform closed-form	Uniform bootstrap	Firths	LASSO	Repeat CV LASSO	Ridge	Repeat CV ridge
1	0.87 (0.68, 1.12)	0.97 (0.73, 1.32)	0.98 (0.75, 1.32)	0.90 (0.71, 1.15)	1.01 (0.77, 1.42)	1.02 (0.77, 1.40)	1.02 (0.79, 1.42)	1.03 (0.79, 1.41)
2	0.89 (0.73, 1.12)	0.99 (0.79, 1.32)	0.99 (0.79, 1.28)	0.91 (0.74, 1.14)	1.07 (0.80, 1.49)	1.08 (0.81, 1.49)	1.03 (0.81, 1.37)	1.03 (0.81, 1.38)
3	0.86 (0.69, 1.13)	0.96 (0.74, 1.32)	0.98 (0.77, 1.32)	0.90 (0.71, 1.17)	1.02 (0.79, 1.36)	1.02 (0.79, 1.39)	1.03 (0.81, 1.43)	1.03 (0.81, 1.42)
4	0.89 (0.74, 1.14)	0.99 (0.80, 1.34)	0.99 (0.81, 1.32)	0.90 (0.76, 1.17)	1.08 (0.84, 1.54)	1.08 (0.86, 1.53)	1.03 (0.83, 1.42)	1.04 (0.83, 1.41)
5	0.91 (0.77, 1.12)	0.98 (0.82, 1.22)	0.98 (0.83, 1.23)	0.93 (0.79, 1.14)	1.01 (0.82, 1.30)	1.02 (0.83, 1.29)	1.02 (0.88, 1.28)	1.02 (0.88, 1.28)
6	0.89 (0.71, 1.15)	1.00 (0.77, 1.39)	0.99 (0.78, 1.34)	0.90 (0.72, 1.16)	1.09 (0.80, 1.55)	1.10 (0.81, 1.57)	1.04 (0.81, 1.44)	1.04 (0.80, 1.45)
7	0.93 (0.79, 1.11)	0.99 (0.83, 1.20)	1.00 (0.84, 1.20)	0.95 (0.81, 1.13)	1.02 (0.84, 1.27)	1.02 (0.84, 1.25)	1.04 (0.91, 1.26)	1.04 (0.91, 1.25)
8	0.90 (0.75, 1.12)	0.99 (0.81, 1.27)	0.99 (0.81, 1.26)	0.92 (0.76, 1.14)	1.09 (0.83, 1.45)	1.07 (0.84, 1.44)	1.03 (0.84, 1.36)	1.03 (0.84, 1.32)

The median (across the 500 iterations for each simulation scenario) of the AUC and Cox-Snell $R^{2}$ , upon validation, were almost identical across methods within each simulation scenario (Supplemental Tables 3 and 4).

#### Distribution of estimated performance upon validation

**Figure 1.**
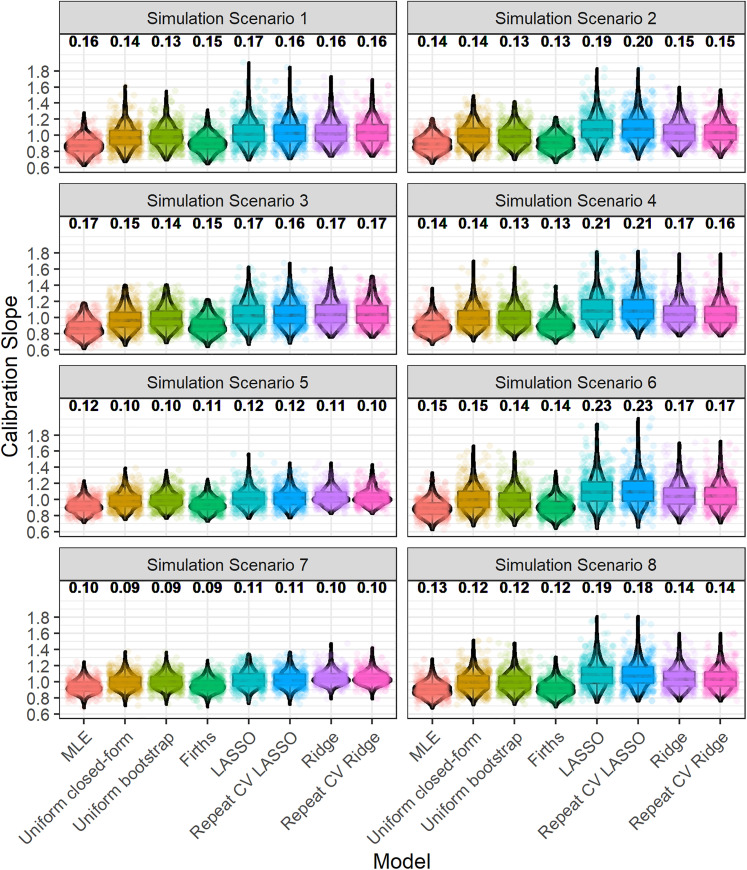
Boxplot and violin plot showing the distribution, across iterations, of the calibration slope, upon validation. The numbers above each plot show the root-mean-square deviation of the calibration slope. Random jitter has been applied to each point to aid visual clarity. The numbering of simulation scenarios is given in Table 1.

Figure 1 depicts the distribution of the calibration slope, upon validation, across iterations. The median interquartile range for calibration slope (across iterations and methods) was approximately 0.12 (with this varying slightly by simulation scenario). The degree of variability in calibration slope (across all methods) was slightly higher in simulation scenarios where the model development sample size calculation was based on the population $R^{2}$ , compared with using 15% of maximum $R^{2}$ (simulation scenarios 1–4 vs 5–8). This was likely driven by the fact that the required sample size using 15% of maximum $R^{2}$ was higher than that based on the population $R^{2}$ .

**Figure 2.**
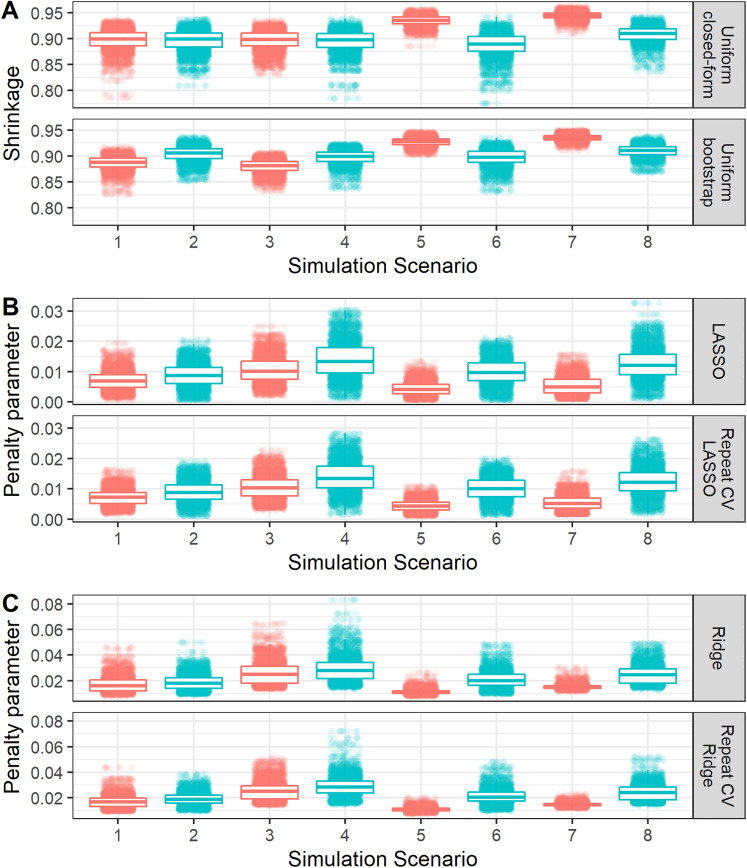
Boxplot and violin plot showing the distribution, across iterations of each simulation scenario, of the shrinkage factor or penalisation terms. Random jitter has been applied to each point to aid visual clarity. The numbering of simulation scenarios is given in Table 1. The colouring on the plot differentiates simulation scenarios where all 10 variables where true predictors (red) or where only 5 of them where true predictors (blue).

As discussed above, the penalisation/shrinkage methods further mitigate the risks of overfitting on average (e.g. Table 2) compared with maximum likelihood. However, by also examining variability in predictive performance, we see from Figure 1 that this comes at the cost of slightly higher variability in predictive performance, upon validation, for LASSO and Ridge compared with maximum likelihood. Specifically, the root-mean-square deviation in calibration slope for the LASSO or Ridge regression was usually slightly higher than (or in some situations equal to) that of maximum likelihood. This is due to the added uncertainty in the underlying shrinkage factor/penalisation estimate (Figure 2). Interestingly, the root-mean-square deviation (variability) in calibration slope for uniform bootstrap shrinkage was consistently lower than that for maximum likelihood, likely because the variability in the estimated shrinkage factor of this method was generally quite low (Figure 2).

These results show the added information that is supplied by exploring variability in performance (and tuning parameters) over just examining average performance. The results suggest that if there is larger uncertainty in the estimates of shrinkage factors or penalisation terms then this corresponds to a higher chance that the model will be miscalibrated, upon independent validation. Therefore, even in data that meet minimum sample size requirements, in practice it will be important to examine the potential uncertainty of penalisation, and therefore predictive performance when developing a CPM (e.g. using bootstrapping; see section ‘Empirical study’). Presenting boxplots as illustrated in this paper would be an informative way of reporting such variability.

Similarly, there was some variability in other performance metrics (calibration-in-the-large, AUC, Cox-Snell $R^{2}$ and Brier score), upon validation, although this was mostly modest and was similar between the estimation methods (Supplemental Figures 2–5). As with calibration, the variability in AUC, Cox-Snell $R^{2}$ and Brier score was generally higher in simulation scenarios where the sample size calculation was based on the population $R^{2}$ (equation (2)), compared with 15% of maximum $R^{2}$ (equation (3)), and where there was more uncertainty in the shrinkage factor/ penalisation estimate (Figure 2).

#### Comparisons in variability of shrinkage and penalisation estimates across methods

As discussed above, investigating the variability across iterations (or, in practice, across bootstrap samples – see section ‘Empirical study’) in the estimates of shrinkage factors or penalisation terms (tuning parameters) is important. Figure 2 shows how the variability compares between methods to conduct each type of shrinkage/penalisation method. Specifically, Figure 2, Panel A shows that the uniform bootstrap method generally resulted in lower variability in the shrinkage factor than the uniform closed-form approach. Figure 2, Panel B compares approaches to undertaking LASSO regression, where the use of repeated 10-fold cross-validation reduced the variability in the penalisation term (λ) compared with single 10-fold cross-validation. Similar findings where observed for Ridge regression (Figure 2, Panel C). These findings agree with previous work.^23^

Across all methods, variability was generally lower when all 10 predictor terms ‘truly’ associated with the outcome or when the sample size calculation was based on maximum $R^{2}$ (i.e. equation (3); that is, simulation scenarios 5–8). Variability was generally higher in scenarios where only five of the predictors ‘truly’ associated with the outcome, especially for LASSO regression.

## Empirical study

In this section, we apply the estimation methods to a real-world critical care example and use bootstrap internal validation to illustrate how one should obtain an indication of variability (uncertainty) in predictive performance in practice, by repeating each modelling step including estimation of the tuning parameters (where relevant).

### Data source, study population and outcomes

De-identified critical care data were obtained from the Medical Information Mart for Intensive Care III (MIMIC-III) database.^38^ MIMIC-III contains information from the Beth Israel Deaconess Medical Center in Boston, Massachusetts, between 2001 and 2012. For this case study, we considered the development of a prediction model for in-hospital mortality after admission to an intensive care unit (ICU). Note the aim was not to develop a CPM for clinical use in this setting, but to illustrate the estimation methods on a real-world dataset, and how one should obtain an indication of variability (uncertainty) in predictive performance in practice.

We defined an ICU admission to be any admission that lasted at least 24 h, and we took the end of day 1 on ICU as the time point at which a prediction is made. We extracted a cohort of patients over 18 years of age, who were admitted to ICU for any cause for at least 24 h. We excluded any ICU admission of less than 24 h. For simplicity, we only included a patient's first ICU admission and first recorded hospitalisation within MIMIC-III.

For the included patients, we extracted information on their age, gender, ethnicity, type of admission, and mean of the lab tests recorded over the first 24 h. Lab tests included measures of the following: bicarbonate, creatinine, chloride, haemoglobin, platelet count, potassium, partial thromboplastin time, international normalized ratio, prothrombin time, blood urea nitrogen and white blood count.

The SQL code to extract the data from the MIMIC-III database is available at https://github.com/GlenMartin31/Penalised-CPMs-In-Minimum-Sample-Sizes.

### Model development and bootstrap internal validation

We developed CPMs for the binary outcome of in-hospital mortality using each of the methods outlined in section ‘Shrinkage and penalisation methods to developing prediction models’. We did not consider predictor selection (with the exception of LASSO, where this is implicit in the method), and all of the models included the following candidate predictors: age (categories of 10-year increments available in MIMIC-III), sex (male vs female), admission type (elective vs non-elective), ethnicity (categorical), and the 24h mean of each of the aforementioned lab tests (all continuous). We considered a total of 23 predictor parameter (accounting for multiple factor levels, where applicable). We undertook a complete case analysis to develop the models; while in practice one should consider alternative approaches to handle missing data, we consider complete case here for illustrative simplicity and computational ease.

We calculated the minimum required sample size to develop a logistic regression model for in-hospital mortality, using the Riley et al. criteria.^13^ The pre-specification of the Cox-Snell $R^{2}$ was made based on 15% of the maximum $R^{2}$ (i.e. equation (3)) using the observed outcome proportion.

We undertook two analyses: first, developing each of the CPMs in the whole MIMIC-III cohort; second, developing each of the CPMs in a random subset of the MIMIC-III cohort with size equal to the minimum required sample size according to the Riley et al. criteria.^13^ Here, the second analysis (hereto called the sub-analysis) is mimicking a situation where the available data exactly matches minimum requirements. In both cases, we applied bootstrap internal validation to assess adjusted calibration and discrimination. Specifically, we took 100 bootstrap samples (with replacement) of the development dataset (either the full cohort or the sub-analysis), applied the *exact same* modelling steps in each bootstrap sample, and calculated the optimism for each performance statistic: that is, the difference between the predictive performance of the models within each bootstrap sample and the predictive performance of each bootstrap CPM applied to the original development data. We then subtracted each of the 100 optimism estimates from the apparent performance (performance of the models developed on MIMIC-III, within the MIMIC-III data) to give 100 optimism-adjusted performance estimates. From these, we summarised both the mean optimism-adjusted performance and visualized the distribution across the 100 bootstraps (to investigate variability, mimicking the simulation above). Bootstrap corrected 95% confidence intervals for each optimism-adjusted performance metric were calculated as the 2.5th and 97.5th percentiles (across the 100 optimism-adjusted performance estimates); an alternative (computationally expensive) approach has been described previously.^39^

### Empirical study results

After applying the inclusion and exclusion criteria, our extracted cohort included 28,859 patients, of which 3316 (11.5%) died in-hospital. Using this observed outcome proportion and 15% of the maximum $R^{2}$ , resulted in a minimum required sample size of 2590, which was driven by criteria 1 of Riley et al.^13^ Thus, the whole MIMIC-III development cohort substantially surpassed the minimum required sample size. A random 2590 samples were selected from the full MIMIC-III dataset for the sub-analysis.

Table 3 shows the mean (taken across the 100 bootstrap samples) optimism-adjusted performance results for each modelling approach, for both the main analysis and the sub-analysis. As expected, each of the models are well calibrated, with the exception of ridge regression which is slightly over-shrunk (calibration slope slightly higher than 1); importantly, the mean calibration slope of the unpenalised MLE model was ≥0.9 , as expected based on the Riley et al. criteria.^13–15^ As with the simulation study, the use of penalisation methods further mitigated against potential overfitting, on average, in these data that met (or surpassed) minimum requirements.

**Table 3.** The mean (95% bootstrap confidence interval) of the optimism-adjusted performance results in the MIMIC-III example for each estimation method. Main study corresponds to model fitting on the whole MIMIC-III dataset, while subset corresponds to the sub-analysis on the minimum required sample size.

Study	Model	Calibration-in-the-large	Calibration slope	AUC	Brier score
Main	MLE	0.00 (−0.04, 0.04)	0.99 (0.94, 1.03)	0.74 (0.73, 0.75)	0.09 (0.09, 0.09)
Main	Uniform closed-form	0.00 (−0.04, 0.04)	1.00 (0.95, 1.04)	0.74 (0.73, 0.75)	0.09 (0.09, 0.09)
Main	Uniform bootstrap	0.00 (−0.04, 0.04)	1.00 (0.96, 1.05)	0.74 (0.73, 0.75)	0.09 (0.09, 0.09)
Main	Firths	0.00 (−0.04, 0.04)	0.99 (0.95, 1.03)	0.74 (0.73, 0.75)	0.09 (0.09, 0.09)
Main	LASSO	0.00 (−0.04, 0.04)	0.99 (0.95, 1.04)	0.74 (0.73, 0.75)	0.09 (0.09, 0.09)
Main	Repeat CV LASSO	0.00 (−0.04, 0.04)	0.99 (0.95, 1.04)	0.74 (0.73, 0.75)	0.09 (0.09, 0.09)
Main	Ridge	0.00 (−0.04, 0.04)	1.05 (1.00, 1.10)	0.74 (0.73, 0.75)	0.09 (0.09, 0.09)
Main	Repeat CV ridge	0.00 (−0.04, 0.04)	1.05 (1.00, 1.10)	0.74 (0.73, 0.75)	0.09 (0.09, 0.09)
Main	LASSO1SE	0.00 (−0.04, 0.04)	1.21 (1.15, 1.27)	0.74 (0.73, 0.75)	0.09 (0.09, 0.10)
Main	Ridge1SE	0.00 (−0.04, 0.04)	1.26 (1.20, 1.32)	0.74 (0.73, 0.75)	0.09 (0.09, 0.10)
Subset	MLE	−0.01 (−0.14, 0.12)	0.90 (0.74, 1.05)	0.74 (0.71, 0.76)	0.10 (0.09, 0.10)
Subset	Uniform closed-form	−0.01 (−0.13, 0.11)	0.99 (0.82, 1.16)	0.74 (0.71, 0.76)	0.10 (0.09, 0.10)
Subset	Uniform bootstrap	−0.01 (−0.13, 0.11)	1.02 (0.84, 1.19)	0.74 (0.71, 0.76)	0.10 (0.09, 0.10)
Subset	Firths	−0.01 (−0.13, 0.11)	0.92 (0.78, 1.06)	0.74 (0.71, 0.76)	0.10 (0.09, 0.10)
Subset	LASSO	−0.01 (−0.13, 0.11)	0.98 (0.83, 1.12)	0.74 (0.71, 0.76)	0.10 (0.09, 0.10)
Subset	Repeat CV LASSO	−0.01 (−0.13, 0.11)	0.99 (0.84, 1.14)	0.74 (0.71, 0.76)	0.10 (0.09, 0.10)
Subset	Ridge	−0.01 (−0.13, 0.11)	1.06 (0.91, 1.21)	0.74 (0.71, 0.76)	0.10 (0.09, 0.10)
Subset	Repeat CV ridge	−0.01 (−0.13, 0.11)	1.04 (0.89, 1.19)	0.74 (0.71, 0.76)	0.10 (0.09, 0.10)
Subset	LASSO1SE	−0.01 (−0.12, 0.10)	1.40 (1.14, 1.67)	0.73 (0.71, 0.76)	0.10 (0.09, 0.11)
Subset	Ridge1SE	−0.01 (−0.12, 0.10)	1.83 (1.53, 2.13)	0.74 (0.71, 0.77)	0.10 (0.09, 0.11)

**Figure 3.**
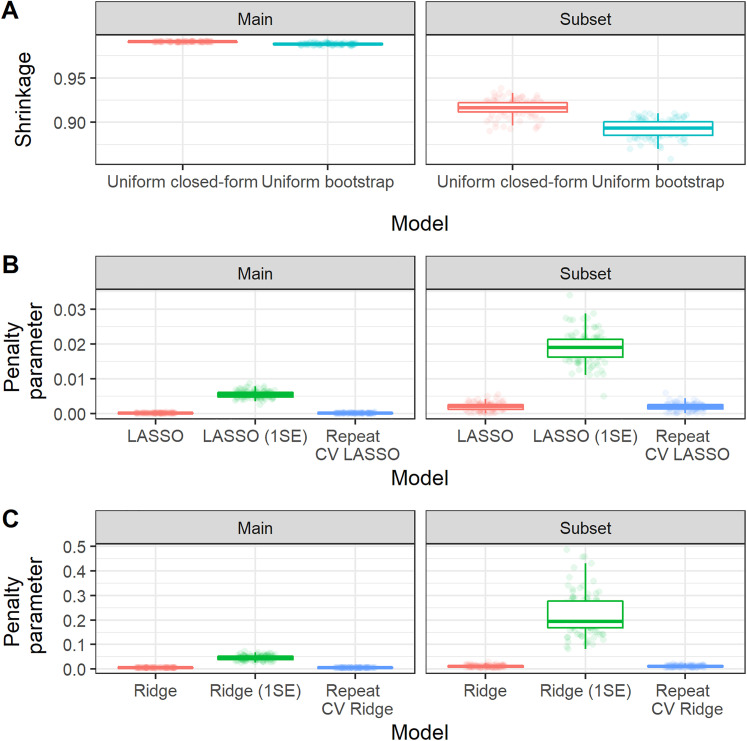
Boxplot showing the distribution, across bootstrap iterations of the MIMIC-III analysis, of the estimated shrinkage factor or penalisation terms. Random jitter has been applied to each point to aid visual clarity. Main study corresponds to model fitting on the whole MIMIC-III dataset, while subset corresponds to the sub-analysis on the minimum required sample size.

**Figure 4.**
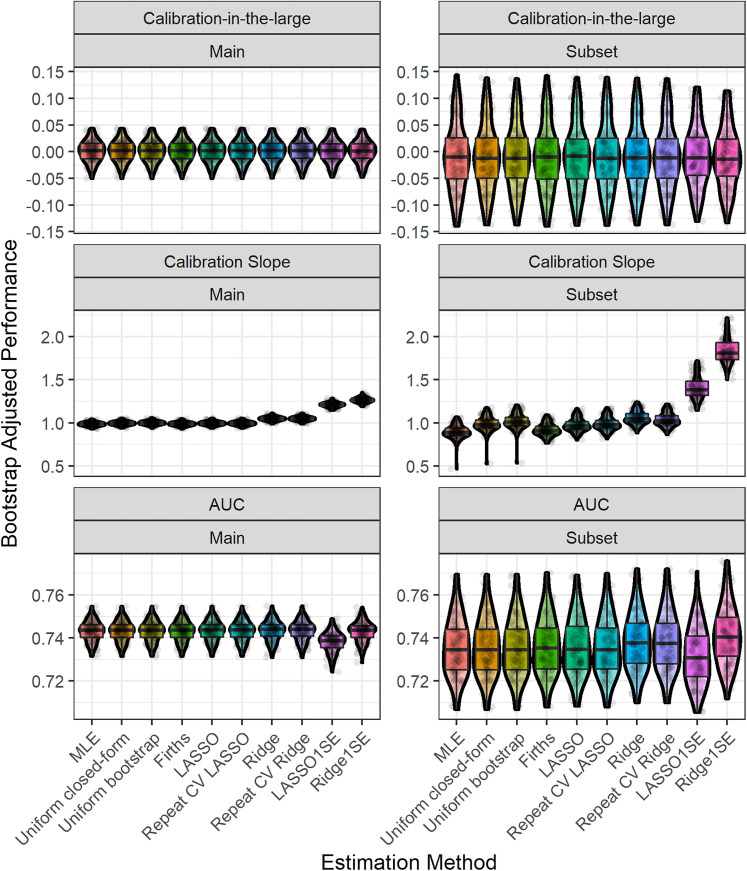
Boxplot and violin plot showing the distribution, across bootstrap iterations, of the (bootstrap) optimism-adjusted performance results in the MIMIC-III example for each estimation method. Random jitter has been applied to each point to aid visual clarity. Main study corresponds to model fitting on the whole MIMIC-III dataset, while subset corresponds to the sub-analysis on the minimum required sample size.

Figure 3 shows the distribution of the estimated shrinkage factors/penalisation terms (tuning parameters) across bootstrap samples. We found that there was larger variability in the shrinkage factors/penalisation terms for the subset analysis than the main analysis (due to the smaller sample size in the former).^23^ Larger variability in the shrinkage factors/penalisation terms resulted in corresponding larger variability in the calibration slope of these methods (Figure 4). For this empirical study, we considered choosing the penalisation term based on the 1-standard-error method for LASSO and Ridge; we found that this generally resulted in larger variability and also lead to underfitting (Figure 4). The width of the 95% bootstrap confidence intervals of each performance metric was larger for the sub-analysis compared with the main analysis (Table 3), caused by larger variability in the distribution of the apparent performance minus optimism across the 100 bootstrap samples (Figure 4). For the main analysis, we found very low levels of variability across bootstrap samples, which is because of the large sample size for model development (relative to the minimum required sample size based on the Riley et al. criteria). For example, in the main analysis, the majority of (adjusted) calibration slope estimates for unpenalised MLE were between 0.95 and 1.05; this gives strong reassurance that the developed CPM will perform well when applied to new individuals from the same population as was used for model development. In contrast, the majority of (adjusted) calibration slope estimates for unpenalised MLE were between 0.8 and 1.1 in the sub-analysis, demonstrating wider variability and hence less reassurance that the developed CPM will perform well when applied to new individuals from the same population as was used for model development.

## Discussion

This study has investigated the predictive performance of CPMs developed in sample sizes that adhere to minimum requirements. We found that, on average, all of the methods resulted in well-calibrated CPMs within an independent dataset, with penalisation/shrinkage further reducing the level of overfitting compared to unpenalised methods. However, this benefit of the penalisation methods came at the cost of slightly increased variability in the performance metrics across simulated/bootstrap datasets; this was often marginal but may still be important in practice. Models that exhibit less variability (uncertainty) in their predictive performance (and their estimated penalty and shrinkage factors) are more likely to correspond to robust CPMs when applied in new individuals. Given these findings, we recommend the use of penalisation/shrinkage methods to develop a CPM within data that (at least) meet minimum sample size criteria,^13–15^ to further help mitigate overfitting, while also examining/reporting the variability in predictive performance (and tuning parameters) as part of the model development process, to help gauge the model's stability, and thus its reliability in new data. This can be achieved by deriving confidence intervals via bootstrap internal validation^39^ and/or plotting the distribution of predictive performance (and tuning parameters) in a similar way to shown in this study.

This study builds upon, and supplements, previous work in this area.^12,16,17,22–24^ Most of the previous literature has focused on the effect of penalisation methods to develop CPMs in terms of varying EPP values. However, following the publication of formal sample size requirements for CPMs,^13–15^ investigating the effect of penalisation methods in data that meet such minimum requirements is crucial. Indeed, contrary to common beliefs, penalisation approaches are not a solution to insufficient sample sizes (or low EPP), especially given the high variability in the effect of penalisation in low sample size settings.^23,24^ This study is the first to investigate variability of performance in data that meet (or surpass) formal minimum sample size requirements.

Some of the findings of this study are, perhaps, unsurprising. Given that we focused on the case of development data that (at least) adhered to minimum sample size requirements, it is unsurprising that MLE resulted in CPMs that were reasonably well calibrated, on average. For example, our use of the Riley et al. sample size criteria targeted a shrinkage of 0.9, so we would naturally expect the mean calibration slope to be ≥0.9 . Nevertheless, one important finding from this study is that the average calibration slope was closer to one for the CPMs developed using penalisation/shrinkage methods, as compared with standard (unpenalised) MLE. These findings illustrate that there are still benefits to applying post-estimation shrinkage or penalised regression methods, within data that meet/surpass minimum sample size requirements, to further help mitigate the risk of overfitting. However, this potentially comes at the price of increased variability in predictive performance for these shrinkage/penalisation methods, compared with MLE, because of the uncertainty in estimating shrinkage factor/penalisation term (e.g. λ ).^23^ As such, one needs to show the variability/uncertainty in the shrinkage factor/penalisation term (λ) , across bootstrap samples. In practice, higher levels of variability should cause greater concern that the model might not work in particular instances within new individuals. Variability (in both predictive performance and estimated shrinkage/penalisation terms) is rarely reported when developing a CPM. Since average performance can be suitable, but could have wide variability, reporting the level of variability adds additional information to supplement the average (optimism-adjusted) performance that is commonly reported.

We found that the level of variability in performance metrics was lower than in previous work,^23,24^ but was still relatively high in some situations. For example, we found that variability was higher in the simulation scenarios where only 5 of the 10 simulated predictor variables where ‘truly’ associated with the outcome (i.e. simulation scenarios 2, 4, 6 and 8), likely caused by the increase ‘noise’ within the dataset. This was particularly apparent for LASSO compared with the other methods we considered, which might be explained by the fact that this is the only method (out of those considered) that incorporates variable selection into the estimation process. We also observed more variability in performance results in the situations where we used the ‘true’ (population-level) Cox-Snell $R^{2}$ (equation (2)) to calculate the minimum sample sizes in the simulation study (i.e. simulation scenarios 1–4). As discussed above, this is a result of the smaller sample sizes (due to larger anticipated Cox-Snell $R^{2}$ ), thereby leading to the larger variability in predictive performance (and estimates of shrinkage factor/penalisation terms), as shown previously.^23^

In this paper, we have illustrated how one can use bootstrap internal validation to understand the likely variability in performance metrics, within the given population. Specifically, each modelling step is repeated during bootstrap internal validation process, including estimation of the tuning parameters (where relevant). When bootstrap internal validation is implemented (as recommended^6,29^), it is common for the point estimates of predictive accuracy to be adjusted by the bootstrap-based optimism estimate, but confidence intervals are not usually corrected.^39^ We recommend that future CPM studies should show the distribution of ‘apparent performance minus each bootstrap optimism estimate’ alongside average (optimism-adjusted) performance. To do so, one can create boxplots of the distribution of adjusted predictive performance (and shrinkage factors/penalisation terms, as relevant) across the bootstrap samples, similar to the graphics presented in this paper. If such plots show that the developed CPM exhibits ‘large’ variability/scatter in calibration (across bootstrap samples), then this would indicate caution about using the CPM within the given population, and flag the need for additional validation (and potential model updating or recalibration), even if average (optimism-corrected) performance is deemed satisfactory in the bootstrap process. What is considered to be ‘large’ variability in predictive performance will be context specific, but (for example) if one finds that average (optimism adjusted) calibration slope is approximately 1 (e.g. using penalisation methods within data that meet minimum requirements), but the (adjusted) calibration slope estimates across bootstrap samples are commonly outside of 0.9–1.1, then this would indicate caution. Moreover, the number of bootstrap samples will affect the amount of variability; hence, we recommend that bootstrap internal validation is undertaken with a large number of samples (e.g. 500, and certainly ≥100 ) and should equally follow best practice recommendations.^6,29^

Our simulation study and the sub-analysis of the empirical study considered development data that met, but did not surpass, minimum sample sizes, while our main empirical study illustrated a case where the size of the development data clearly surpassed minimum requirements. We note that usually one would strive for larger samples than a minimum threshold. In the main empirical study, the larger sample sizes (relative to minimum requirements) reduced the variability in tuning parameters and in predictive performance compared with the simulation and empirical sub-analysis. Thus, if one wished to strive for narrower variability, then larger-than-minimum sample sizes would be required, or one would need to calculate the sample size formula under more stringent criteria (e.g. increase the shrinkage factor from 0.9 to 0.95^13,14^). Similarly, the findings from our simulation study where we used the ‘true’ (population-level) Cox-Snell $R^{2}$ (equation (2)) to calculate the minimum sample sizes, showed that if this *a priori* value was not achieved by the model upon validation, then this can cause the average calibration slope to drop below the targeted 0.9. This is an expected analytical property of the sample size formula^13,14^ and indicates that it can be beneficial to be conservative when selecting the anticipated Cox-Snell $R^{2}$ for the sample size calculations. Indeed, our results indicate that being more stringent with the sample size formula (i.e. increasing minimum sample size requirements) would likely lead to reduced variability in predictive performance upon validation (and estimates of shrinkage factor/penalisation terms^23,24^), which in turn would increase confidence that the model will perform well upon validation in new data from the same population as was used to develop the model. Such information is shown explicitly through our recommendations to report/visualise the variability when developing a CPM (additional to only reporting mean performance, which will be adequate if minimum sample size calculations have been adhered to). However, larger sample sizes will not always be achievable in all clinical contexts, which gives further motivation for the need to report variability in predictive performance to supplement average (optimism-adjusted) performance results.

A number of limitations should be considered when interpreting the findings of this study. First, while our empirical data illustrated situations where development data met or surpassed minimum requirements, the generalisability of the empirical findings needs to be considered. Second, we did not consider choices that modellers might need to make when developing a CPM (e.g. variable selection, missing data imputation or consideration of interaction terms), which might increase the level of variability in performance within independent data. This practical point adds further emphasis for the need for those developing CPMs to report/show the variability in performance. Third, we only considered CPMs developed using logistic regression, and continuous or time-to-event outcomes were not explored; however, we would not expect the results to differ substantially. Finally, all of the models in our simulation and empirical analyses had AUC values between 0.7 and 0.8. In practice, CPMs might have AUC values lower than this. We note, however, that if the ‘true’ AUC was lower than those considered in the study, then this would effectively mean the ‘true’ R2 was lower (e.g. see Riley et al.^40^), which in turn would increase the minimum required sample size. Despite this being sample size being larger in absolute terms, it would still be the minimum required for that particular situation, so there will still be variability; again, reporting this variability will directly show this, irrespective of the (average) performance of the CPM.

In conclusion, the use of penalisation methods can further mitigate risks of overfitting even within datasets that adhere to, or surpass, minimum suggested sample sizes. However, although this might resolve overfitting on average, in a particular dataset it may still not be perfect, and indeed because of the need to estimate tuning parameters (that define shrinkage), it comes at the costs of slightly higher variability in predictive performance. Thus, we recommend the use of penalisation/ shrinkage methods to develop a CPM within data that (at least) meet minimum sample size criteria,^13–15^ to further help mitigate overfitting, while also investigating (and reporting) variability in predictive performance through robust bootstrap internal validation, including accounting for the uncertainty in estimating shrinkage/tuning parameters. Those models that exhibit less variability (uncertainty) in their predictive performance (and their estimated tuning parameters/shrinkage factors) are more likely to correspond to robust CPMs when applied in new individuals.

## Supplemental Material

sj-pdf-1-smm-10.1177_09622802211046388 - Supplemental material for Developing clinical prediction models when adhering to minimum sample size recommendations: The importance of quantifying bootstrap variability in tuning parameters and predictive performance Supplemental material, sj-pdf-1-smm-10.1177_09622802211046388 for Developing clinical prediction models when adhering to minimum sample size recommendations: The importance of quantifying bootstrap variability in tuning parameters and predictive performance by Glen P Martin, Richard D Riley, Gary S Collins and Matthew Sperrin in Statistical Methods in Medical Research
