# Supplementary material for: Developing clinical prediction models when adhering to minimum sample size recommendations: The importance of quantifying bootstrap variability in tuning parameters and predictive performance
Source: Stat Methods Med Res. 2021 Oct 8;30(12):2545–61. doi: 10.1177/09622802211046388 (PMC8649413; doi:10.1177/09622802211046388)
Supplement: sj-pdf-1-smm-10.1177_09622802211046388 - Supplemental material for Developing clinical prediction models when adhering to minimum sample size recommendations: The importance of quantifying bootstrap variability in tuning parameters and predictive performance [file sj-pdf-1-smm-10.1177_09622802211046388.pdf]

Supplementary Material

Developing Clinical Prediction Models when adhering to minimum sample size recommendations: the importance of quantifying bootstrap variability in tuning parameters and predictive performance

Glen P. Martin; Richard D. Riley; Gary S. Collins; Matthew Sperrin

Supplementary Tables

Supplementary Table 1: Summary of the minimum required sample sizes, across each simulation scenario. See Table 1 in the main paper for the numbering of each simulation scenario

Simulation Scenario	median	mean	min	max	Sample Size Criteria
1	551	551	544	559	Uniquely criteria 1
2	970	970	952	990	Uniquely criteria 1
3	385	385	385	385	Uniquely criteria 3
4	655	655	645	666	Uniquely criteria 1
5	898	898	898	898	Uniquely criteria 1
6	898	898	898	898	Uniquely criteria 1
7	749	749	749	749	Uniquely criteria 1
8	749	749	749	749	Uniquely criteria 1

Supplementary Table 2: The median (2.5% and 97.5% quantile) of the calibration-in-the-large for each analytical method, upon validation, across the 500 iterations for each simulation scenario. See Table 1 in the main paper for the numbering of each simulation scenario

**Simulation Scenario**	**MLE**	**Uniform closed-form**	**Uniform bootstrap**	**Firths**	**LASSO**	**Repeat CV LASSO**	**Ridge**	**Repeat CV Ridge**
1	-0.01 (-0.23, 0.27)	-0.01 (-0.22, 0.26)	-0.01 (-0.22, 0.25)	-0.01 (-0.23, 0.26)	0.00 (-0.23, 0.25)	0.00 (-0.22, 0.25)	-0.01 (-0.22, 0.25)	-0.01 (-0.22, 0.25)
2	0.00 (-0.16, 0.17)	0.00 (-0.17, 0.17)	0.00 (-0.17, 0.17)	0.00 (-0.16, 0.17)	0.00 (-0.17, 0.16)	0.00 (-0.17, 0.16)	0.00 (-0.17, 0.17)	0.00 (-0.17, 0.17)
3	-0.01 (-0.25, 0.24)	-0.01 (-0.23, 0.23)	-0.01 (-0.23, 0.23)	-0.01 (-0.24, 0.23)	-0.01 (-0.23, 0.22)	-0.01 (-0.23, 0.23)	-0.01 (-0.23, 0.23)	-0.01 (-0.22, 0.23)
4	0.00 (-0.18, 0.17)	0.00 (-0.17, 0.17)	0.00 (-0.17, 0.17)	0.00 (-0.18, 0.17)	0.00 (-0.17, 0.17)	0.00 (-0.16, 0.18)	0.00 (-0.17, 0.17)	0.00 (-0.17, 0.17)
5	0.00 (-0.17, 0.20)	0.00 (-0.17, 0.19)	0.00 (-0.17, 0.19)	0.00 (-0.17, 0.19)	0.00 (-0.17, 0.19)	0.00 (-0.17, 0.19)	0.00 (-0.17, 0.19)	0.00 (-0.17, 0.19)
6	0.00 (-0.17, 0.18)	0.00 (-0.17, 0.18)	0.00 (-0.17, 0.18)	0.00 (-0.17, 0.18)	0.00 (-0.16, 0.17)	0.00 (-0.16, 0.17)	0.00 (-0.16, 0.18)	0.00 (-0.16, 0.18)
7	0.00 (-0.17, 0.15)	0.00 (-0.16, 0.15)	0.00 (-0.16, 0.15)	0.00 (-0.17, 0.15)	0.00 (-0.16, 0.15)	0.00 (-0.16, 0.15)	0.00 (-0.16, 0.15)	0.00 (-0.16, 0.15)
8	0.01 (-0.14, 0.17)	0.01 (-0.13, 0.16)	0.01 (-0.13, 0.16)	0.01 (-0.13, 0.16)	0.00 (-0.13, 0.16)	0.00 (-0.13, 0.16)	0.01 (-0.13, 0.16)	0.01 (-0.13, 0.16)

Supplementary Table 3: The median (2.5% and 97.5% quantile) of the Cox-Snell R2 for each analytical method, upon validation, across the 500 iterations for each simulation scenario. See Table 1 in the main paper for the numbering of each simulation scenario

**Simulation Scenario**	**MLE**	**Uniform closed-form**	**Uniform bootstrap**	**Firths**	**LASSO**	**Repeat CV LASSO**	**Ridge**	**Repeat CV Ridge**
1	0.13 (0.11, 0.14)	0.14 (0.12, 0.15)	0.14 (0.12, 0.15)	0.14 (0.12, 0.14)	0.14 (0.12, 0.14)	0.14 (0.12, 0.15)	0.14 (0.12, 0.15)	0.14 (0.12, 0.15)
2	0.08 (0.07, 0.08)	0.08 (0.07, 0.09)	0.08 (0.07, 0.09)	0.08 (0.07, 0.09)	0.08 (0.07, 0.09)	0.08 (0.07, 0.09)	0.08 (0.07, 0.09)	0.08 (0.07, 0.09)
3	0.19 (0.16, 0.20)	0.19 (0.17, 0.20)	0.19 (0.17, 0.20)	0.19 (0.16, 0.20)	0.19 (0.17, 0.20)	0.19 (0.17, 0.20)	0.19 (0.17, 0.20)	0.19 (0.17, 0.20)
4	0.12 (0.10, 0.12)	0.12 (0.10, 0.12)	0.12 (0.10, 0.12)	0.12 (0.10, 0.12)	0.12 (0.11, 0.12)	0.12 (0.11, 0.12)	0.12 (0.10, 0.12)	0.12 (0.10, 0.12)
5	0.14 (0.13, 0.15)	0.14 (0.13, 0.15)	0.14 (0.13, 0.15)	0.14 (0.13, 0.15)	0.14 (0.13, 0.15)	0.14 (0.13, 0.15)	0.14 (0.13, 0.15)	0.14 (0.13, 0.15)
6	0.08 (0.07, 0.08)	0.08 (0.07, 0.09)	0.08 (0.07, 0.08)	0.08 (0.07, 0.08)	0.08 (0.07, 0.09)	0.08 (0.07, 0.09)	0.08 (0.07, 0.08)	0.08 (0.07, 0.08)
7	0.20 (0.19, 0.21)	0.20 (0.19, 0.21)	0.20 (0.19, 0.21)	0.20 (0.19, 0.20)	0.20 (0.19, 0.20)	0.20 (0.19, 0.20)	0.20 (0.19, 0.21)	0.20 (0.19, 0.21)
8	0.12 (0.10, 0.12)	0.12 (0.11, 0.12)	0.12 (0.11, 0.12)	0.12 (0.10, 0.12)	0.12 (0.11, 0.12)	0.12 (0.11, 0.12)	0.12 (0.11, 0.12)	0.12 (0.11, 0.12)

Supplementary Table 4: The median (2.5% and 97.5% quantile) of the AUC for each analytical method, upon validation, across the 500 iterations for each simulation scenario. See Table 1 in the main paper for the numbering of each simulation scenario

**Simulation Scenario**	**MLE**	**Uniform closed-form**	**Uniform bootstrap**	**Firths**	**LASSO**	**Repeat CV LASSO**	**Ridge**	**Repeat CV Ridge**
1	0.76 (0.75, 0.77)	0.76 (0.75, 0.77)	0.76 (0.75, 0.77)	0.76 (0.75, 0.77)	0.76 (0.75, 0.77)	0.76 (0.75, 0.77)	0.76 (0.75, 0.77)	0.76 (0.75, 0.77)
2	0.70 (0.69, 0.71)	0.70 (0.69, 0.71)	0.70 (0.69, 0.71)	0.70 (0.69, 0.71)	0.70 (0.69, 0.71)	0.70 (0.69, 0.71)	0.70 (0.69, 0.71)	0.70 (0.69, 0.71)
3	0.75 (0.74, 0.76)	0.75 (0.74, 0.76)	0.75 (0.74, 0.76)	0.75 (0.74, 0.76)	0.76 (0.74, 0.76)	0.76 (0.74, 0.76)	0.75 (0.74, 0.76)	0.75 (0.74, 0.76)
4	0.70 (0.69, 0.70)	0.70 (0.69, 0.70)	0.70 (0.69, 0.70)	0.70 (0.69, 0.70)	0.70 (0.69, 0.70)	0.70 (0.69, 0.70)	0.70 (0.69, 0.70)	0.70 (0.68, 0.70)
5	0.77 (0.76, 0.77)	0.77 (0.76, 0.77)	0.77 (0.76, 0.77)	0.77 (0.76, 0.77)	0.77 (0.76, 0.77)	0.77 (0.76, 0.77)	0.77 (0.76, 0.77)	0.77 (0.76, 0.77)
6	0.70 (0.69, 0.71)	0.70 (0.69, 0.71)	0.70 (0.69, 0.71)	0.70 (0.69, 0.71)	0.70 (0.69, 0.71)	0.70 (0.69, 0.71)	0.70 (0.69, 0.71)	0.70 (0.69, 0.71)
7	0.76 (0.75, 0.76)	0.76 (0.75, 0.76)	0.76 (0.75, 0.76)	0.76 (0.75, 0.76)	0.76 (0.75, 0.76)	0.76 (0.75, 0.76)	0.76 (0.75, 0.76)	0.76 (0.75, 0.76)
8	0.70 (0.69, 0.70)	0.70 (0.69, 0.70)	0.70 (0.69, 0.70)	0.70 (0.69, 0.70)	0.70 (0.69, 0.70)	0.70 (0.69, 0.70)	0.70 (0.69, 0.70)	0.70 (0.69, 0.70)

Supplementary Figures

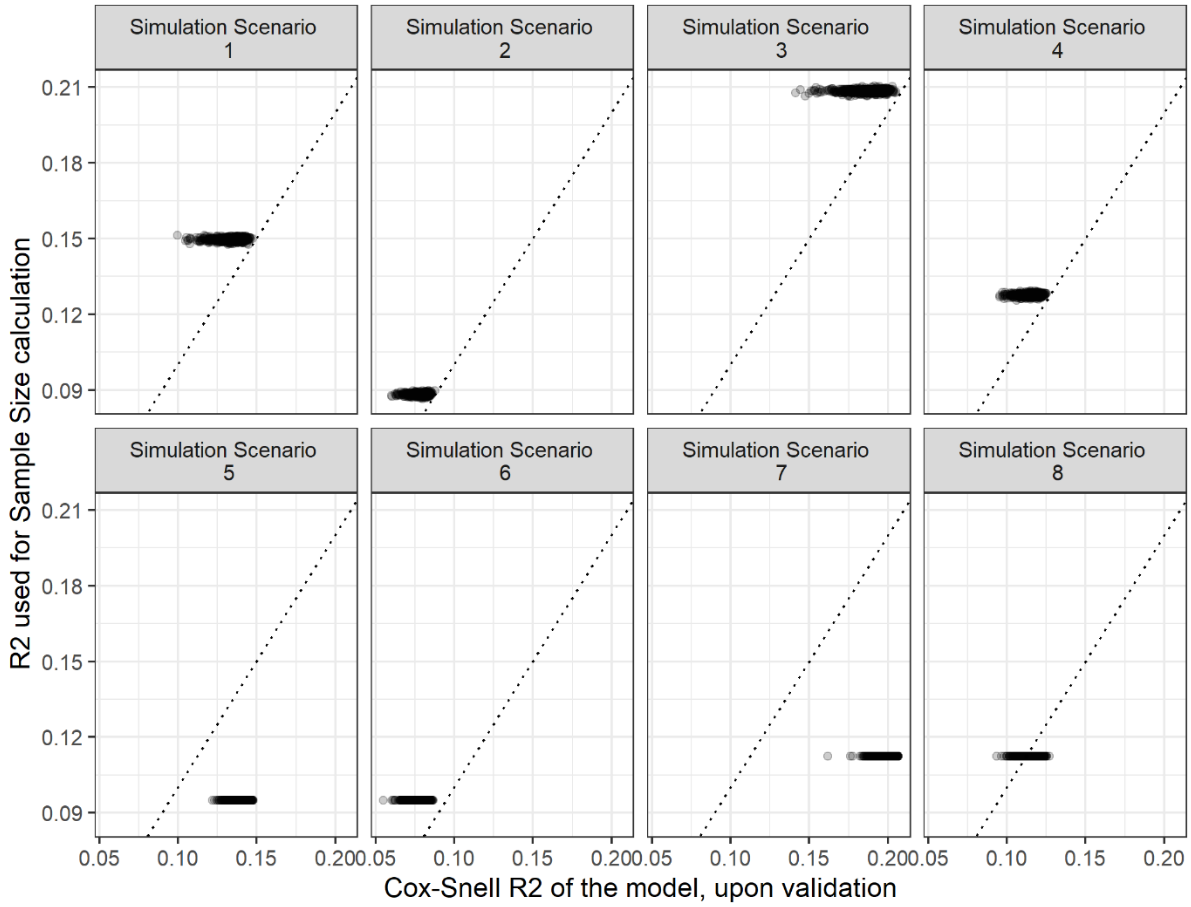

Supplementary Figure 1: Scatter plots of the Cox-Snell R2 that was used within the sample size calculations for the simulation study, against the Cox-Snell R2 achieved for the prediction models estimated by MLE, upon validation. Each panel represents a given simulation scenario; the numbering of simulation scenarios is given in Table 1.

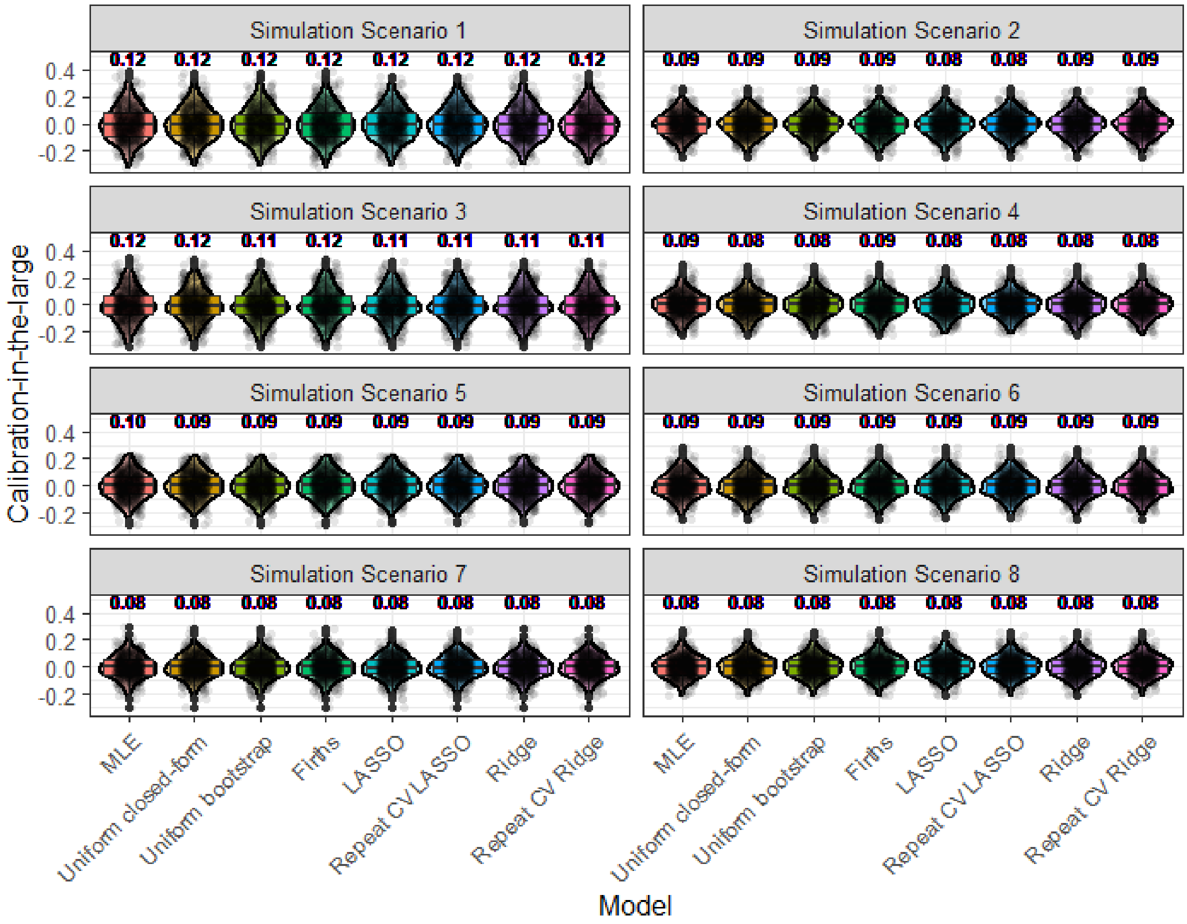

Supplementary Figure 2: Boxplot and violin plot showing the distribution, across iterations, of the calibration-in-the-large, upon validation. The numbers above each plot show the root-mean-square deviation of the calibration-in-the-large. Random jitter has been applied to each point to aid visual clarity. The numbering of simulation scenarios is given in Table 1.

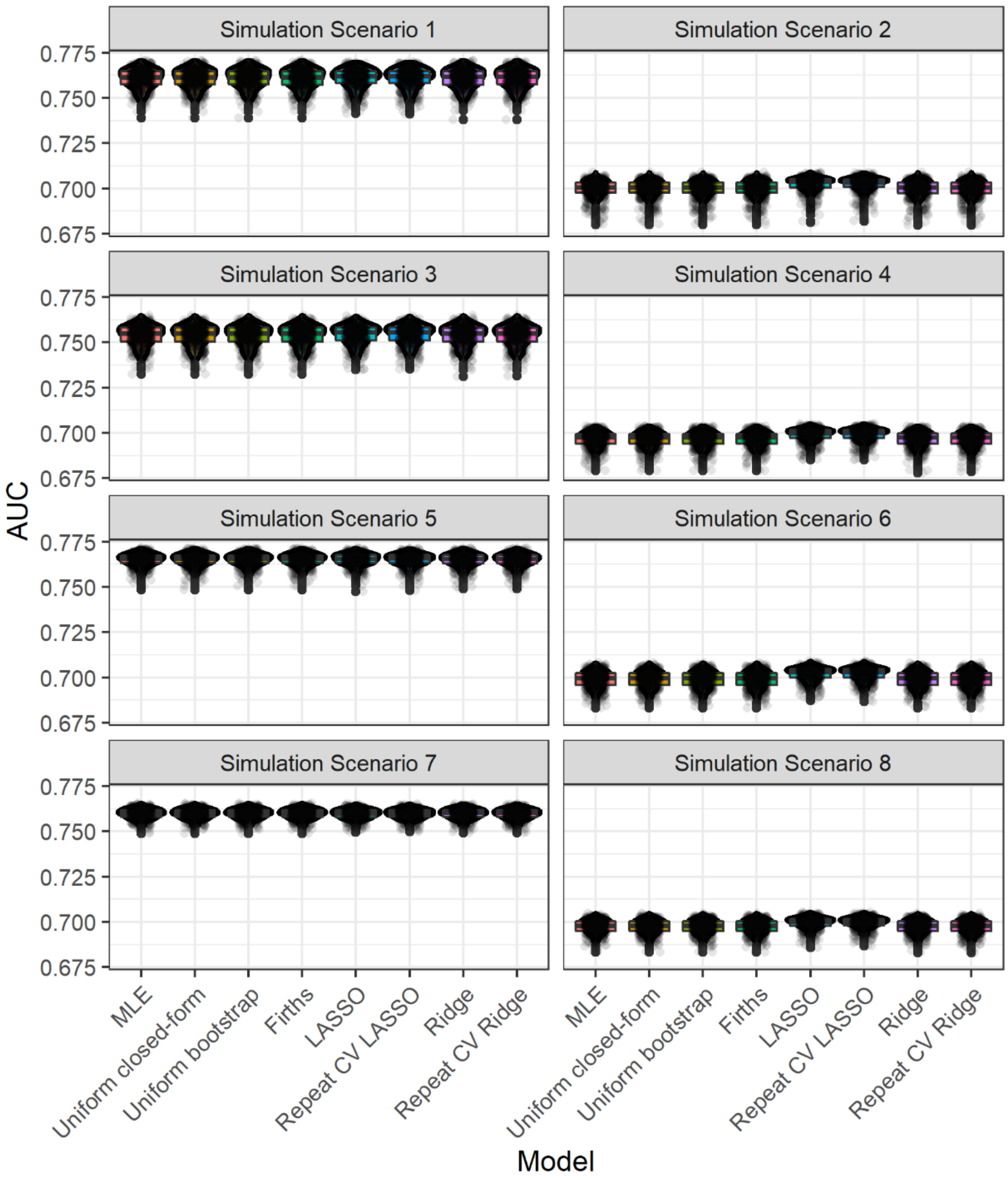

Supplementary Figure 3: Boxplot and violin plot showing the distribution, across iterations, of the AUC, upon validation. Random jitter has been applied to each point to aid visual clarity. The numbering of simulation scenarios is given in Table 1.

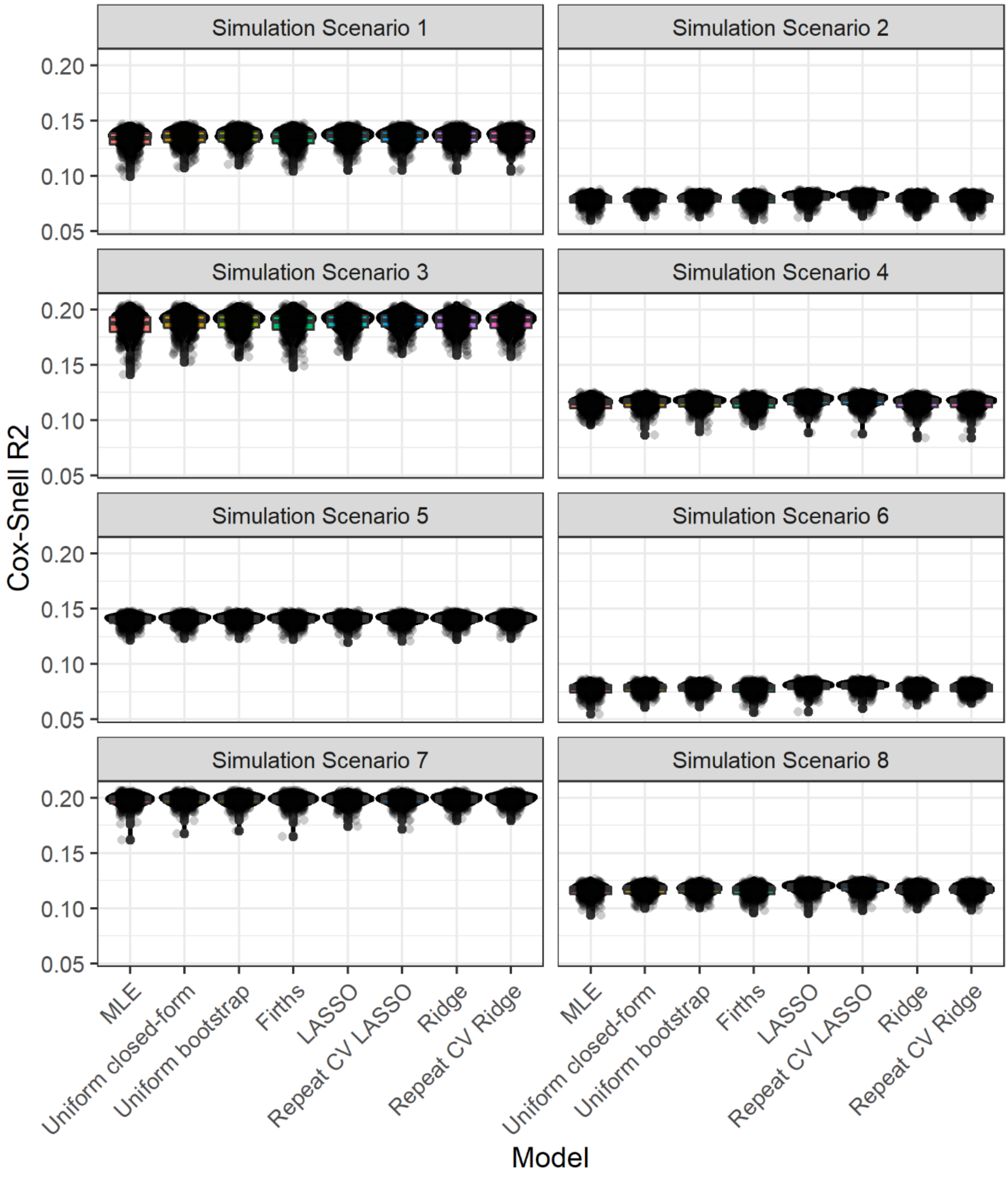

Supplementary Figure 4: Boxplot and violin plot showing the distribution, across iterations, of the Cox-Snell R2, upon validation. Random jitter has been applied to each point to aid visual clarity. The numbering of simulation scenarios is given in Table 1.

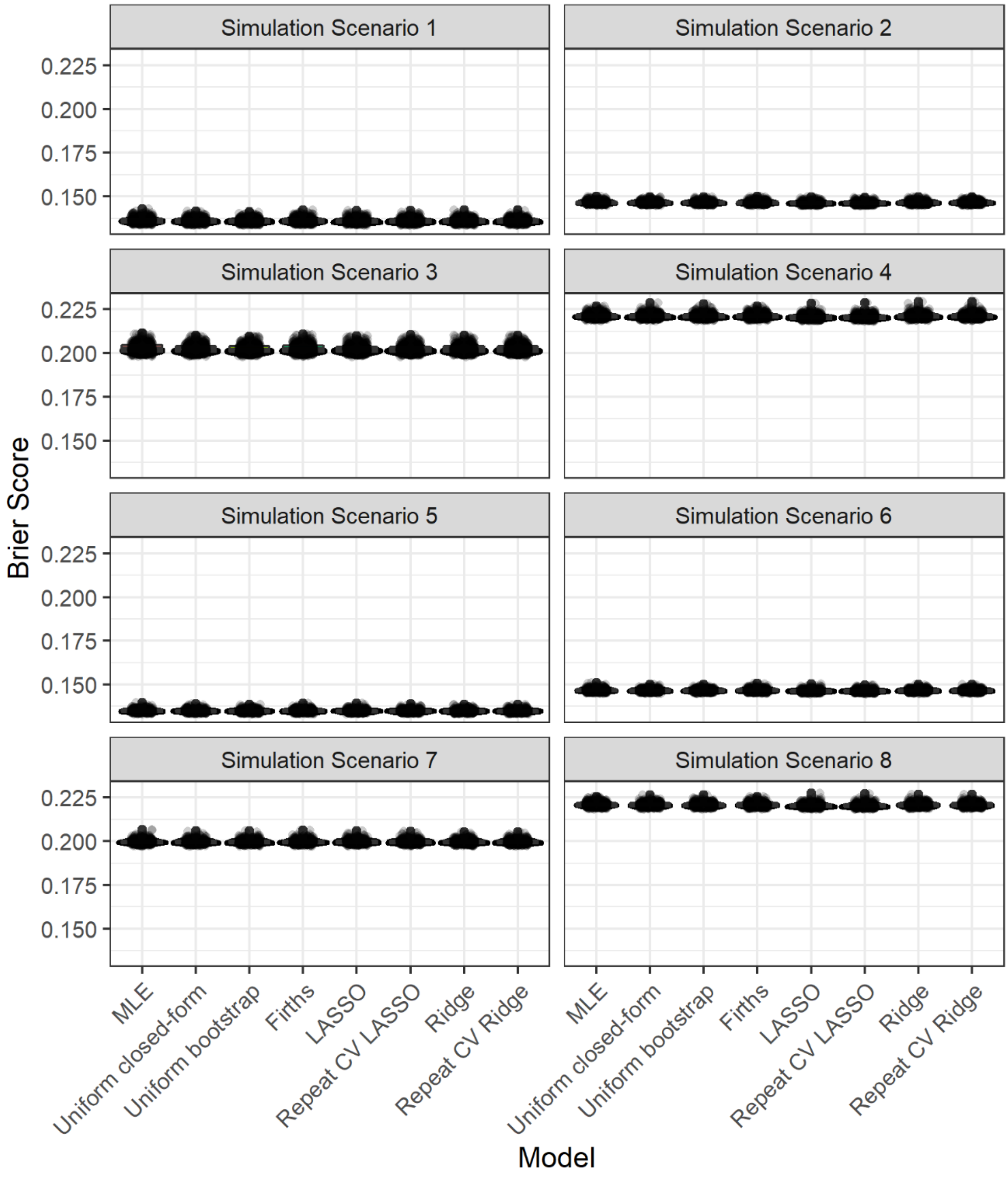

Supplementary Figure 5: Boxplot and violin plot showing the distribution, across iterations, of the Brier Score, upon validation. Random jitter has been applied to each point to aid visual clarity. The numbering of simulation scenarios is given in Table 1.
